# Evaluation of Various Nuclear Cytological Changes in Normal Buccal Mucosa and Peritumoural Area in Patients with Oral Squamous Cell Carcinoma Receiving Concomitant Chemoradiotherapy

**DOI:** 10.1155/2016/6293795

**Published:** 2016-04-11

**Authors:** Sadia Minhas, Muhammad Kashif, A. H. Nagi

**Affiliations:** ^1^Department of Oral Pathology, Akhtar Saeed Medical and Dental College, Bahria Town, Lahore 54000, Pakistan; ^2^Department of Immunology, University of Health Sciences, Lahore, Pakistan; ^3^Department of Morbid Anatomy and Histopathology, University of Health Sciences, Lahore, Pakistan

## Abstract

*Objectives*. To evaluate the role of serial cytological assay in calculating the nuclear response of contralateral normal buccal mucosa and peritumoural area of squamous cell carcinoma of oral cavity in patients receiving fractionated radiotherapy (RT) and chemotherapy.* Materials and Methods*. This prospective, nonrandomized study was comprised of 76 histologically confirmed cases of oral squamous cell carcinoma on cyclical chemoradiation treatment. Chemoradiosensitivity was evaluated using serial scrape smears taken before and after immediate exposure to CCRT, at 17th day of CCRT (mid of treatment), and at the end of treatment. The nuclear changes, such as multinucleation, micronucleation, karyorrhexis, karyolysis, nuclear budding, prominent nucleoli, and binucleation occurring in both irradiated cancer cells and contralateral normal buccal mucosa, had a statistically significant dose related increase with concomitant chemoradiotherapy (*p* < 0.05).* Conclusion*. We recommend regular use of serial cytological assay during CCRT as it may prove to be a valuable tool for assessment of chemoradiosensitivity and persistence of tumour/dysplastic cells after radiotherapy.

## 1. Introduction

Globally, oral cancer is the eighth most common cause of cancer-related deaths, although many people are unaware of its presence [1]. Of these oral cancers, more than 90% are oral squamous cell carcinomas (OSCC) arising in the mucous membranes of the oral cavity, floor of the mouth, ventral surface of the tongue, and oropharynx [2]. A study conducted by Bhurgi in Pakistan, reported that it is the most common cancer in Pakistan, whereas Rehman and Jafferi found that oral carcinoma constitutes about 10% of the malignancies in Pakistan [3]. The early oral cancers are usually treated with surgery and radiation therapy, whereas patients with advanced oral tumours may undergo combination of treatments, that is, radiation therapy, chemotherapy, and concomitant chemoradiotherapy. The selection of treatments depends on patient general health, the size of tumour, and metastasis [4]. The radiation which is used for the treatment of cancers is ionizing radiation because it produces ions in the cells of the tissue from where it passes. This can kill cells or alter genes so that the cells cannot grow [5].

The drugs flow all-round the body in the blood and reach cancer cells nearly anywhere in the body. This is called systemic treatment. Chemotherapy drugs deteriorate the genes inside the nucleus of cells. A combination of different chemotherapy drugs are used for the treatment of cancer. The combination of chemotherapy drugs damages cells at different stages in the course of cell division. There is more chance of killing more cells with the use of more than one type of drug [6]. A concurrent chemoradiotherapy (CCRT) regimen represents the most excellent current standard therapy for many patients with regionally advanced solid tumours and improves the likelihood of cure. The clinical goal of administrating chemotherapy and radiation concurrently is to develop both locoregional and systemic tumour control [7].

For oral cancer, cytology has been an option and proved to be a consistent primary diagnostic test. It can also be of value where surgical biopsy is not indicated or in postradiotherapy follow-up cases. The combined histological and cytological assessment of a lesion has been found to give the highest percentage of early diagnosis of oral cancers [8]. Cytological effects of radiation on oral mucosa and in oral cancers were reported in 1957 and 1959, respectively [9]. Numerous reports have described different cytoplasmic and nuclear changes following radiation therapy. These changes consist of cytoplasmic granulation, cellular enlargement, vacuolization, pyknosis, binucleation, karyorrhexis, karyolysis, micronucleation, nuclear budding, nuclear enlargement, and multinucleation [10].

By the 1960s, the nuclear morphological alterations that were calculated by cytology became well established and included karyorrhexis, karyolysis, multinucleation, and crenation of nuclear membrane [11]. Despite a high incidence of oral cancer in Asian region, studies on prediction of effectiveness of various treatment modalities are inadequate and sparse, in this region [12]. The present study was taken forward to see whether serial cytological evaluation from normal buccal mucosa and peritumoural area in oral squamous cell carcinoma patients receiving concomitant chemoradiotherapy at four different stages can predict the chemoradiosensitivity of peritumoural area and response of normal buccal mucosa.

## 2. Materials and Methods

The study was conducted in Department of Morbid Anatomy and Histopathology, University of Health Sciences Lahore Pakistan, during the period of one year. A total of 76 patients with histologically proven squamous cell carcinoma of oral cavity, on fractionated radiotherapy on a dose of 70–90 Gy in 5 fractions/week for a span of 7 weeks (total 35 fractions), were included in the study. Scrape smears were collected from peritumoural area (the peritumoural area is defined as a 2 mm wide band of host tissue adjacent to the invasive front. The tumour periphery is defined as a 2 mm wide band of tumour immediately adjacent to the invasive front, and tumour center is defined as an inner center area) and contralateral normal buccal mucosa of each patient before the start, after immediate exposure to CCRT, on 17th day, and at the end of treatment. The cytological smears were obtained by scraping the peritumoural area and contralateral normal buccal mucosa using the rounded end of Ayre's spatula and slides were fixed in 95% alcohol for haematoxylin and eosin and Papanicolaou (Pap) stains, while some slides were air dried for May-Grunwald-Giemsa (MGG) stain. Smears were examined at both 20x and 40x with an eyepiece of 10x. Around 500–1000 cells were evaluated from the samples collected on each occasion. The nuclear changes were observed, that is, multinucleation, nuclear budding, karyolysis, karyorrhexis, micronuclei, binucleation, and prominent nucleoli. Data were entered and analyzed using the SPSS 20.0. The variation in the frequency of these changes in relation to cumulative radiation dose was analyzed using Chi-square test/Fisher exact test and a *p* value < 0.05 was considered as statistically significant. The study was approved and certified by Institutional Ethical Review Committee and Advanced Studies & Research Board, UHS Lahore, Pakistan.

## 3. Results

In 304 smears from normal buccal mucosa on all respective days of CCRT, karyolysis was seen in *n* = 156 (51.3%) smears and it was absent in *n* = 148 (48.7%) smears. Out of 76 smears from normal buccal mucosa on each day, that is, before and immediately after first dose, at 17th day, and at end of CCRT, karyolysis was seen in *n* = 1 (1.3%), *n* = 3 (3.9%), and *n* = 76 (100%) for 17th day and end of therapy smears, respectively. In overall 304 smears from peritumoural area on all respective days of sampling, karyolysis was seen in *n* = 180 (59.2%) smears and it was absent in *n* = 124 (40.8%) smears.

Considering the karyolysis in peritumoural area before and after immediate exposure to CCRT it was seen in *n* = 7 (9.2%) and *n* = 21 (27.6%) smears, respectively. Smears were taken on each day, that is, at the 17th day and at the end of therapy; all the smears (100%) were positive for karyolysis.

Destructive fragmentation of nucleus (karyorrhexis) in normal buccal mucosal cells on all particular days of treatment from 76 patients was observed in *n* = 154 (50.6%) smears and absent in *n* = 150 (49.3%) smears. Among 76 smears from normal buccal mucosa on all sampling days, that is, before and after immediate exposure, at 17th day, and at end of CCRT, karyorrhexis was observed in 1.3%, 2.6%, 98.7%, and 100% smears, respectively.

While inspecting the 304 smears from peritumoural area on all specific days of CCRT, karyorrhexis was seen in *n* = 174 (57.2%) smears. In *n* = 130 (42.7%) smears no karyorrhexis was seen in peritumoural area. Out of 76 smears from peritumoural area on each day, that is, before CCRT *n* = 7 (9.2%), after immediate exposure *n* = 15 (9.7%), at 17th day, and at end of CCRT, *n* = 76 (100%) of smears were positive for karyorrhexis (Table 1).

**Figure 1:**
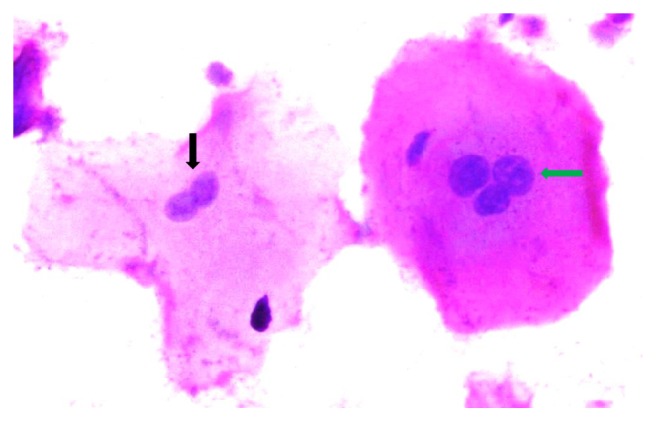
Photomicrograph with H&E stain shows multinucleated cell (green arrow) and binucleated cell (black arrow) in smears from contralateral normal buccal mucosa obtained at end of CCRT.

Regarding binucleation in contralateral normal buccal mucosal cells, it was present in *n* = 190 (62.5%) out of 304 smears while it was absent in *n* = 114 (37.5%) smears. A total of 76 smears from normal buccal mucosa before CCRT *n* = 7 (9.2%), after immediate exposure to CCRT *n* = 32 (42.1%), at the 17th day of treatment 98.7%, and at the end of treatment *n* = 76 (100%) smears were positive for binucleation (Figure 1).

Among all the 304 smears from peritumoural on all particular days of sampling, binucleation was observed in *n* = 263 (86.5%) and absent in *n* = 41 (13.5%) smears (Figure 1). On each day of sampling, binucleation was observed in *n* = 49 (64.5%) before CCRT, after immediate exposure to CCRT in *n* = 63 (82.9%), at the 17th day of treatment in 98.7% (Figure 1), and at the end of CCRT in 100% of smears (Table 1).

Considering the nuclear budding in normal buccal mucosal cells on all specific days of CCRT, it was present in *n* = 90 (29.6%) smears and was absent in *n* = 214 (70.3%) smears. From normal buccal mucosa on each specific day, that is, before and after immediate exposure to CCRT none of the smears showed nuclear budding. However, at the 17th day and at the end of treatment (Figure 5) nuclear budding was observed in *n* = 16 (21.1%) and *n* = 74 (97.4%) smears, respectively.

In a total of 304 smears from peritumoural area from 76 patients on all respective days of treatment, nuclear budding was observed in *n* = 105 (34.5%) smears and absent in *n* = 199 (65.4%) smears. Before and after immediate exposure to CCRT nuclear budding was observed in *n* = 1 (1.3%) smears for both days of therapy. Whereas *n* = 28 (36.8%) smears were taken at the 17th day of treatment and at the end of treatment *n* = 75 (98.7%) smears were positive for nuclear budding.

Regarding micronucleation in a total of 304 smears from normal buccal mucosa on all specific days of sampling, it was seen in *n* = 173 (56.9%) smears and absent in *n* = 131 (43.1%) smears. Out of 76 smears from normal buccal mucosa, before CCRT *n* = 3 (3.9%) and after immediate exposure to treatment *n* = 18 (23.7%) smears were positive for micronucleation. However, in smears taken on each day, that is, at the 17th day of therapy and at the end of therapy, 100% of smears were positive for micronucleation.

In a total of 304 smears from peritumoural area on all particular days of treatment, micronucleation was observed in *n* = 222 (73.0%) smears. However it was absent in *n* = 82 (26.9%) smears. In a total of 76 smears taken at each specific day of CCRT, that is, before CCRT, after immediate exposure to CCRT, at the 17th day of CCRT (Figure 3), and at end of therapy micronucleation was observed in smears as follows: *n* = 18 (23.7%), *n* = 53 (69.7%), 98.7%, and 100%, respectively (Table 1).

**Figure 2:**
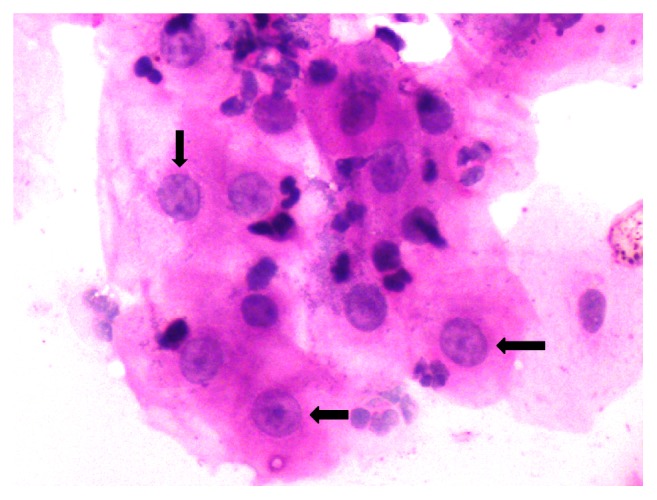
Photomicrograph reveals prominent nucleoli in almost each and every cell in smears obtained from contralateral normal buccal mucosa at 17th day of CCRT.

**Figure 3:**
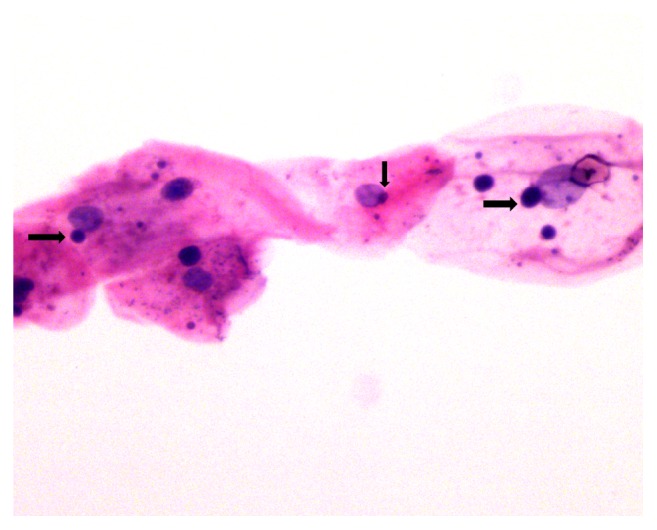
Photomicrograph with H&E stain shows feature of nuclear atypia, that is, micronuclei (arrows) in smears obtained at the 17th day of CCRT from peritumoural area.

Among 304 smears from normal buccal mucosa on all specific days of CCRT from 76 patients, prominent nucleoli were seen in *n* = 225 (74.0%) smears and were absent in *n* = 79 (25.9%) smears. Among 76 smears from normal buccal mucosa on each specific day of sampling, that is, before CCRT and after immediate exposure to CCRT, prominent nucleoli were seen in *n* = 15 (19.7%) and 76.3% smears, respectively, while smears both at 17th day (Figure 2) and at end of therapy were 100% positive for prominent nucleoli.

On peritumoural area, prominent nucleoli were present in *n* = 290 (95.4%) smears and were absent in *n* = 14 (4.6%) smears. Among 76 smears on each day, that is, before and after immediate exposure to CCRT, prominent nucleoli were seen in *n* = 63 (82.9%) and 98.7% smears, whereas prominent nucleoli were observed in 100% smears taken at each day, that is, at the 17th day and end of therapy, respectively, from peritumoural area.

**Figure 4:**
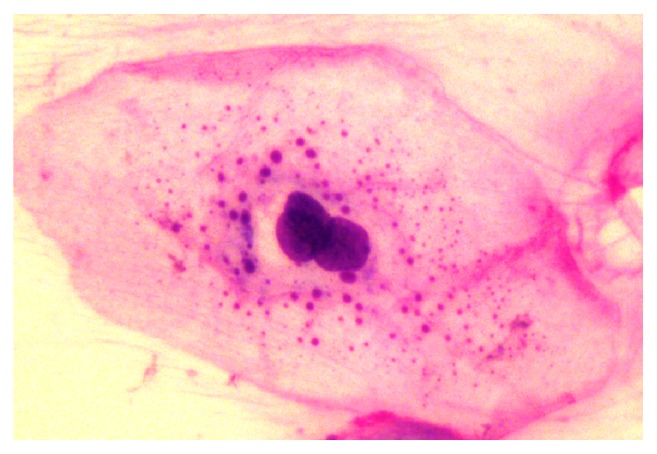
Photomicrograph with H&E stain shows a multinucleated cell in smear obtained from peritumoural area at the 17th day of CCRT.

In normal buccal mucosal cells, multinucleation was observed in *n* = 69 (22.6%) smears on all specific days of sampling from 76 patients. No such changes were seen in *n* = 235 (77.3%) smears (Figure 1). As considering the smears from peritumoural area on all particular days of CCRT, multinucleation was observed in *n* = 93 (30.5%) smears (Figure 4). Detailed distribution of multinucleation both on normal buccal mucosa and on peritumoural area on different days of sampling is given in Table 1.

## 4. Discussion

Oral squamous cell carcinoma is a serious fatal disease. The outcomes from management of advanced carcinoma of the head and neck are unfortunate. The conventional management procedures for SCC of head and neck are radiotherapy and surgery, which are mostly curative in early stages of disease and are less efficient in more advanced patients. Although squamous cell carcinoma of the head and neck is chemosensitive, it is not treatable by chemotherapy alone [13–15]. One method, in an attempt to improve clinical outcomes, is to give radiotherapy and chemotherapy concomitantly [16].

Responses of malignant cells as well as the surrounding normal oral tissues to radiotherapy are based on their radiosensitivity but the inconsistency in host-tumour reaction makes it complicated to evaluate and predict the effect of such treatment in a particular patient. Radiation puts forth its effects on both malignant and normal cells mostly by inducing chromosomal injury, the end result of which can be distinguished by the occurrence of micronuclei in dividing cells [17].

Improvement of the radiation response was usually less marked in the tumour model as compared to normal tissues. The combined drug-radiation effect was actually less time-dependent in the tumour than in the normal tissues [18].

The present study was designed to determine the cytological changes following concomitant chemoradiotherapy (radiochemoreaction) and predict the strength of association among duration and dose of chemoradiation therapy to these changes.

In this study, the changes were evaluated in peritumoural area around the tumour and benign cells collected from contralateral normal buccal mucosa. Our results showed that various quantifiable cellular changes become evident in the initial few days of concomitant chemoradiotherapy but are more prominent at the end of CCRT where they illustrate strong statistical significance (Table 2).

Karyorrhexis is the destructive fragmentation of the nucleus of a dying cell whereby its chromatin is distributed irregularly throughout the cytoplasm. It signifies nuclear breakup into smaller fragments, whereas karyolysis indicates a progressive dissolution of chromatin. Karyolysis and karyorrhexis were reported on both normal and malignant oral cells and also noted a statistically significant increase with radiotherapy dose (*p* = 0.01) [19–21]. Serial cytological smears from OSCC show that both karyorrhexis and karyolysis were more pronounced at the end of therapy [10].

Nuclear budding is defined as production of two daughter nuclei of unequal size by constriction of the parent nucleus. It represents the rounded nuclear material mimicking a micronucleus and can often be found close to the nucleus without any definite separation. Nuclear budding can occur in the nuclear envelope and when it ruptures it leads to transfer of DNA material to cytoplasm. Nuclear budding can be considered to be stimulated by a direct, localized consequence of radiation on nuclear membrane [22, 23]. The frequency of nuclear budding was increased with increased radiotherapy dosages in serial scrape smears from contralateral normal buccal mucosa and malignant tissue, and a statistically significant association has been reported with the radiotherapy dose (*p* < 0.001) on each site (Table 2) [19]. Similarly, serial scrape smears from radiotherapy receiving patients for OSCC reported that statistically significant association was observed between nuclear budding and radiotherapy dosages on tumoural area (*p* = 0.034) (Table 2) [21].

Cell division is accomplished when the splitting up of cell membrane happens subsequent to nuclear division. Incomplete cell division occurs due to cell membrane damage which directs to the development of binucleated and multinucleated cells by recurring nuclear division. Membrane lipids can undergo peroxidation subsequent to irradiation and this injury might be adequate to avoid cell wall division in sensitive cells [24]. Multinucleation (Figure 4) is considered by having more than one nucleus per cell. It is caused by membrane damage related with increased proliferation of the nucleus resulting in lack of ability of the membrane to continue with the nuclear division. Silverman et al. stated that multinucleation is a frequent radiation-induced change in oral cancers and this was accepted by other researchers later on [25, 26]. Radiation stimulated multinucleation has been noted in cell culture and animals experiments. Damage to nuclear membrane has been suggested as a mechanism that directs to cell death; as a result multinucleated cells are considered to be dead cells and unable of giving rise to colonies [27]. Mehrotra and his colleagues observed that in normal mucosa and malignant cells, the frequency of multinucleation was increased with increased radiotherapy dosage in a serial scrape smears from both sites. They also reported a significant association between multinucleation on normal mucosa and malignant cells and radiation dose (*p* < 0.001) [22]. Similarly, the frequency of multinucleated cells in tumoural area in irradiated serial smears was increased with increased radiotherapy dosages [21, 26].

Binucleation is defined as formation of two nuclei within a cell through division of the nucleus without division of the cytoplasm. The study conducted in India on binucleation and radiation response in normal buccal mucosa and malignant cells stated that there was increased incidence of binucleation on both sites as the radiotherapy dose increased. Similarly, a significant association was observed between radiotherapy dose and binucleation on both contralateral normal mucosa and in malignant cells (*p* < 0.001) in present study [22].

Micronucleation is also the name given to the small nucleus that forms whenever a chromosome or a fragment of a chromosome is not incorporated into one of the daughter nuclei during cell division. Micronuclei are intracytoplasmic DNA staining bodies found in the same level as the main nucleus with the equal or a little staining intensity, one-third to one-fifth of the size of the main nucleus placed within two nuclear diameters from the main nucleus but distinctly separated from it. The micronuclei represent the genomic damage of the cell and to calculate the genomic damage as a result of radiotherapy. The micronuclei present in normal buccal mucosa cells are noteworthy, because they are in irradiated field. The existence of micronucleus shows that the cell has undergone through unrepaired DNA damage and the cells containing micronuclei are considered as dead cells that are unable to give rise to progeny [28]. Existence of a micronucleus is a recognized test for observing the toxicity of chemicals in normal tissues and efficiency of chemopreventive agents against cancer [29]. The number of micronucleated cells raises and achieves a plateau with repetitive chemical and radiation injuries [22]. Hintzsche et al. examined normal buccal mucosa cells for genomic damage at four different times during radiation therapy. A clear increase was observed in normal buccal mucosa for every time point [30]. The frequency of MN in oral mucosal cells of patients with OSCC was three- to fourfold higher as compared with the control group [31]. In a study, Dórea and his colleagues stated that incidence of micronuclei was seen considerably more commonly in cells collected from lesions than in cells from normal areas [32]. Similar findings were reflected in our study with the frequency of micronuclei increasing with radiation dose on both sites. Previous multiple studies with multinucleation and micronucleation assays during the first 15–18 days of radiotherapy showed that serial cytology has important and significant correlations with cell proliferation and radiosensitivity [12, 25, 33].

Prominent nucleoli are defined as small, typically round granular bodies composed of protein and RNA in the nucleus of a cell. Nucleoli are usually associated with a specific chromosomal site and involved in ribosomal RNA synthesis and the formation of ribosomes. The study conducted in USA reported that the incidence of prominent nucleoli was raised in smears obtained from benign prostate glands after radiotherapy [34]. A study carried out in Chicago stated that chemotherapy could induce prominent nucleoli [35].

Significant association was observed among different variables of nuclear atypia (karyolysis, karyorrhexis, nuclear budding, micronucleation, prominent nucleoli, binucleation, and multinucleation) and days of CCRT in present study (Table 2).

Another finding which is apparent in the present study is a nonlinear increase in the numbers of nuclear abnormalities on exposure to increasing doses of concomitant chemoradiotherapy. It can be observed from the graphs (Figure 6) that the rise in the frequencies of the different nuclear abnormalities follows a gentle curve up till after immediate exposure to CCRT but after that the rise is relatively much steeper. A similar “hockey stick” curve for stimulation of micronuclei in lymphocytes exposed to ionizing radiation has been stated earlier and might result from DNA repair processes taking place at low radiation doses promoting reattainment of DNA strand breaks at some stage in the S phase of cell cycle prior to the cell entry in mitotic phase [36]. With a raise in the chemoradiation dose the repair ability of the cell decreases while the amount of chromosomal damage increases accelerating to increase in unrepaired DNA fragments which may lead to development of more number of micronuclei and nuclear buddings. An almost identical accumulation of unrepaired damage to the cell membrane at elevated doses of chemoradiation may cause an increase in the counts of multinucleated and binucleated cells too.

All the nuclear abnormalities that were studied, that is, micronucleation, nuclear budding, binucleation, and multinucleation, showed a dose dependent increase in response to concomitant chemoradiotherapy for both sites (contralateral normal buccal mucosa and peritumoural area) and these changes were more marked in CCRT receiving patients as compared to patients receiving radiotherapy alone.

**Figure 5:**
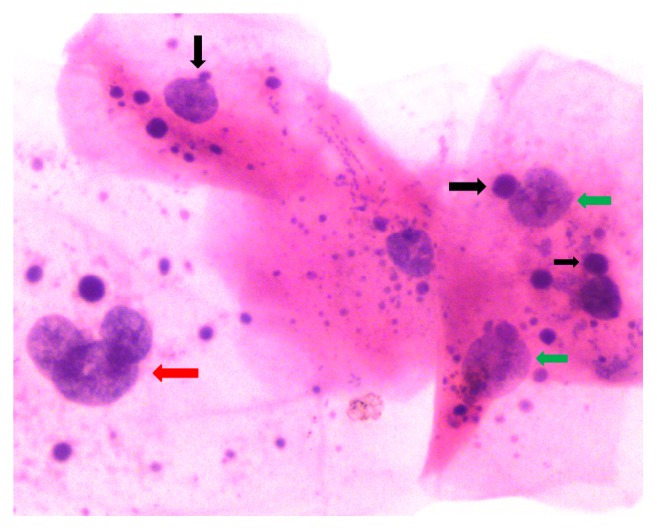
Photomicrograph of smear obtained at the end of CCRT from contralateral normal buccal mucosa shows features of nuclear atypia, micronuclei (black arrow), mild to moderate degree of pleomorphism (green arrow), and nuclear budding (red arrow).

**Figure 6:**
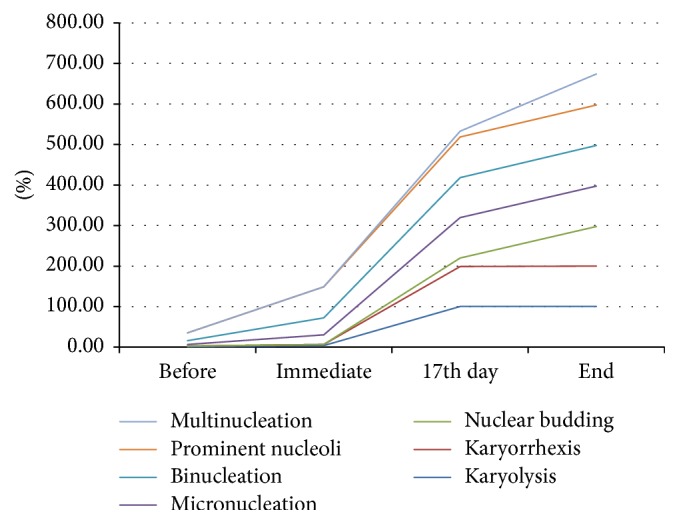
Nonlinear increase among the variables of nuclear atypia and different days of CCRT in smears obtained from contralateral normal buccal mucosa.

These all previously described features, that is, karyolysis, karyorrhexis, binucleation, nuclear budding, and multinucleation, are collectively called nuclear atypia (Figure 5). The study conducted by Bhattathiri called the “abnormal nucleated cells” with these features and many other international studies reported that these features are indicators/markers of radiosensitivity of tumour. It is a useful tool in the assessment of biological damage that can help in radiosensitivity of tumour whereas tumours which are radioresistant exhibited lesser degree of change as compared to radiosensitive tumours [10, 26, 37]. We observed that when different parameters (multinucleated cells, nuclear budding, micronucleation, and bizarre cells) were taken together, the increase in dose related response was significantly high. These abnormally nucleated cells had a mean value four times higher than pretreatment count at 38.5 Gy, thus indicating these combined parameters to be a better indicator of radiosensitivity than any single parameter [37, 38].

## 5. Conclusion

It is concluded from the findings of present study that various nuclear abnormalities reveal a statistically significant increase with increasing chemoradiation doses and time interval. Persistence of dysplastic and malignant cells from peritumoural area during and at end of this treatment can be a sign of resistant or recurrent carcinoma. Similarly, the relationship among different days of CCRT and high frequency of nuclear abnormalities in normal buccal mucosa suggests that the serial smears of these changes have potential use for the early prediction of inflammatory-benign-precancerous and then malignant lesions in patients receiving CCRT. We, therefore, suggest serial cytological assays of peritumoural area and from contralateral normal buccal mucosa till the end of therapy and also on longer term follow-ups.

## Figures and Tables

**Table 1:** Frequencies of different variables of nuclear atypia in cells of peritumoural area on different days of CCRT.

Variables/days of CCRT	Before	Immediate	17th day	End
Karyolysis	9.2%	27.6%	100%	100%
Karyorrhexis	9.2%	9.7%	100%	100%
Binucleation	64.5%	82.9%	98.7%	100%
Multinucleation	0%	0%	30.3%	92.1%
Nuclear budding	1.3%	1.3%	36.8%	98.7%
Micronuclei	23.7%	69.7%	98.7%	100%
Prominent nucleoli	82.9%	98.7%	100%	100%

**Table 2:** Association between days of CCRT and various variables of nuclear abnormalities on both sites (contralateral normal buccal mucosa and peritumoural area).

Variables/site	Buccal mucosa	Peritumoural area
Karyolysis	*p* = 0.000	*p* = 0.000
Karyorrhexis	*p* = 0.000	*p* = 0.000
Multinucleation	*p* = 0.000	*p* = 0.000
Nuclear budding	*p* = 0.000	*p* = 0.000
Prominent nucleoli	*p* = 0.000	*p* = 0.000
Binucleation	*p* = 0.000	*p* = 0.000
Micronuclei	*p* = 0.000	*p* = 0.000

## References

[1] Johnson N. W., Jayasekara P., Amarasinghe A. A., Hemantha K. (2011). Squamous cell carcinoma and precursor lesions of the oral cavity: epidemiology and aetiology. *Periodontology 2000*.

[2] Darby M. L., Walsh M. M. (2010). *Dental Hygiene Theory and Practice*.

[3] Musani M. A., Jawed I., Marfani S., Khambaty Y., Jalisi M., Khan S. A. (2009). Carcinoma cheek: regional pattern and management. *Journal of Ayub Medical College, Abbottabad*.

[4] NCI (2009). *What Do You Need to Know about Oral Cancer?*.

[5] American Cancer Society (2013). *Types of Radiation Used to Treat Cancer. Radiation Therapy Principles*.

[6] Cancer Research UK (2013). *How Chemotherapy Kills Cancer Cells. How Chemotherapy Works*.

[7] Seiwert T. Y., Salama J. K., Vokes E. E. (2007). The chemoradiation paradigm in head and neck cancer. *Nature Clinical Practice Oncology*.

[8] Cowpe J. G., Longmore R. B., Green M. W. (1988). Quantitative exfoliative cytology of abnormal oral mucosal smears. *Journal of the Royal Society of Medicine*.

[9] Rimpu K., Chaugule A., Goyal P. K. (2005). Karyoanomalic frequency during radiation therapy. *Journal of Cancer Research and Therapeutics*.

[10] Bindu L., Balaram P., Mathew A., Remani P., Bhattathiri V. N., Nair M. K. (2003). Radiation-induced changes in oral carcinoma cells—a multiparametric evaluation. *Cytopathology*.

[11] Wachtel E. G. (1964). Radiation changes in vaginal smears. *Exfoliative Cytology in Gynecological Practice*.

[12] Ramaesh T., Ratnatunga N., Mendis B. R., Rajapaksa S. (1998). Exfoliative cytology in screening for malignant and premalignant lesions in the buccal mucosa. *The Ceylon Medical Journal*.

[13] Stell P. M., Dalby J. E., Strickland P., Fraser J. G., Bradley P. J., Flood L. M. (1983). Sequential chemotherapy and radiotherapy in advanced head and neck cancer. *Clinical Radiology*.

[14] Tannock I. F. (1989). Combined modality treatment with radiotherapy and chemotherapy. *Radiotherapy and Oncology*.

[15] Stell P. M., Rawson N. S. B. (1990). Adjuvant chemotherapy in head and neck cancer. *British Journal of Cancer*.

[16] Vokes E. E., Weichselbaum R. R. (1990). Concomitant chemotherapy: rationale and clinical experience in patients with solid tumours. *Journal of Clinical Oncology*.

[17] Sarto F., Tomanin R., Giacomelli L., Iannini G., Cupiraggi A. R. (1990). The micronucleus assay in human exfoliated cells of the nose and mouth: application to occupational exposures to chromic acid and ethylene oxide. *Mutation Research Letters*.

[18] von der Maase H. (1986). Experimental studies on interactions of radiation and cancer chemotherapeutic drugs in normal tissues and a solid tumour. *Radiotherapy and Oncology*.

[19] Mehrotra R., Madhu, Singh M. (2004). Serial scrape smear cytology of radiation response in normal and malignant cells of oral cavity. *Indian Journal of Pathology and Microbiology*.

[20] Mehrotra R., Gupta A., Singh M., Ibrahim R. (2006). Application of cytology and molecular biology in diagnosing premalignant or malignant oral lesions. *Molecular Cancer*.

[21] Agarwal D., Khan N., Siddhiqui S. A., Afroz N. (2011). Assessment of various cytological changes for predicting radiosensitivity of oral cavity cancer by serial cytology. *JK Science*.

[22] Mehrotra R., Goel N., Singh M., Kumar D. (2004). Radiation-related cytological changes in oral malignant cells. *Indian Journal of Pathology and Microbiology*.

[23] Raj V., Mahajan S. (2011). Dose response relationship of nuclear changes with fractionated radiotherapy in assessing radiosensitivity of oral squamous cell carcinoma. *Journal of Clinical and Experimental Dentistry*.

[24] Ogden G. R., McQueen S., Chisholm D. M., Lane E. B. (1993). Keratin profiles of normal and malignant oral mucosa using exfoliative cytology. *Journal of Clinical Pathology*.

[25] Bhattathiri N. V., Bindu L., Remani P., Chandralekha B., Nair K. M. (1998). Radiation-induced acute immediate nuclear abnormalities in oral cancer cells: serial cytologic evaluation. *Acta Cytologica*.

[26] Kumari R., Arun C., Goyal P. K. (2005). Karyoanomalic frequency during radiation therapy. *Journal of Cancer Research and Therapeutics*.

[27] Man Y.-G., Nieburgs H. E. (2006). A subset of cell clusters with malignant features in morphologically normal-appearing and hyperplastic tissues. *Cancer Detection and Prevention*.

[28] Ogden G. R., Cowpe J. G., Wight A. J. (1997). Oral exfoliative cytology: review of methods of assessment. *Journal of Oral Pathology and Medicine*.

[29] Stich H. F., Rosin M. P. (1984). Micronuclei in exfoliated human cells as a tool for studies in cancer risk and cancer intervention. *Cancer Letters*.

[30] Hintzsche H., Polat B., Schewe V. (2012). Micronucleus formation kinetics in buccal mucosa cells of head and neck cancer patients undergoing radiotherapy. *Toxicology Letters*.

[31] Jadhav K., Gupta N., Ahmed Mujib B. R. (2011). Micronuclei: an essential biomarker in oral exfoliated cells for grading of oral squamous cell carcinoma. *Journal of Cytology*.

[32] Dórea L.-N. T. M., Meireles J. R. C., Lessa J. P. R. (2012). Chromosomal damage and apoptosis in exfoliated buccal cells from individuals with oral cancer. *International Journal of Dentistry*.

[33] Sharma P., Kumar N., Bahadur A. K., Mandal A. K. (2005). Ki-67 expression in cytologic scrapes from oral squamous cell carcinoma before and after 24 gray radiotherapy—a study on 43 patients. *Medicina Oral, Patología Oral y Cirugía Bucal*.

[34] Cheng L., Cheville J. C., Bostwick D. G. (1999). Diagnosis of prostate cancer in needle biopsies after radiation therapy. *American Journal of Surgical Pathology*.

[35] Doria M. I., Doria L. K., Faintuch J., Levin B. (1994). Gastric mucosal injury after hepatic arterial infusion chemotherapy with floxuridine: a clinical and pathologic study. *Cancer*.

[36] Mitchell J. C., Norman A. (1987). The induction of micronuclei in human lymphocytes by low doses of radiation. *International Journal of Radiation Biology*.

[37] Bhattathiri N. V., Bharathykkutty C., Prathapan R., Chirayathmanjiyil D. A., Nair K. M. (1998). Prediction of radiosensitivity of oral cancers by serial cytological assay of nuclear changes. *Radiotherapy and Oncology*.

[38] Bhattathiri V. N., Bindu L., Remani P., Chandralekha B., Davis C. A., Nair M. K. (1996). Serial cytological assay of micronucleus induction: a new tool to predict human cancer radiosensitivity. *Radiotherapy and Oncology*.

